# Suppressive effects of dRYamides on feeding behavior of the blowfly, *Phormia regina*

**DOI:** 10.1186/s40851-015-0034-z

**Published:** 2015-12-08

**Authors:** Toru Maeda, Yuki Nakamura, Hajime Shiotani, Masaru K. Hojo, Taishi Yoshii, Takanori Ida, Takahiro Sato, Morikatsu Yoshida, Mikiya Miyazato, Masayasu Kojima, Mamiko Ozaki

**Affiliations:** Department of Biology, Graduate School of Science, Kobe University, Nada, Kobe, 657-8501 Japan; Department of Biology, Graduate School of Natural Science and Technology, Okayama University, Okayama, 700-8530 Japan; Interdisciplinary Research Organization, University of Miyazaki, Miyazaki, 889-2192 Japan; Molecular Genetics, Institute of Life Sciences, Kurume University, Fukuoka, 839-0864 Japan; Department of Biochemistry, National Cerebral and Cardiovascular Center Research Institute, Suita, 565-0873 Japan

**Keywords:** Bioactive peptide, dRYamide, Feeding regulation, Taste response, Fly

## Abstract

Recently, dRYamides-1 and -2 have been identified as ligands of the neuropeptide Y-like receptor CG5811 in *Drosophila melanogaster*. It has also been reported in brief that injection of dRYamide-1suppresses the early feeding behavior called proboscis extension reflex (PER) in the blowfly *Phormia regina*. Immunohistochemical analyses by our group using anti-dRYamide-1 antiserum indicated symmetrical localization of 32 immunoreactive cells in the brain of *P. regina*. In order to analyze the mechanism of feeding regulation, we further investigated the effects of dRYamide-1 and -2 on intake volume, PER exhibition, and activity of the sugar receptor neuron. After injection of dRYamide-1 or -2, flies showed little change in the intake volume of sucrose solution, but a significant depression of PER to sucrose. Injection of dRYamide-1 revealed a significant decrease in the responsiveness of the sugar receptor neuron, although the injection of dRYamide-2 did not. These results suggest that the dRYamide peptides decrease feeding motivation in flies, as evaluated by PER threshold, through a mechanism that partially involves desensitization of the sugar receptor neuron.

**Electronic supplementary material:**

The online version of this article (doi:10.1186/s40851-015-0034-z) contains supplementary material, which is available to authorized users.

## Introduction

Bioactive peptides have been broadly studied in vertebrates and invertebrates [1–4]. Studies in *Drosophila melanogaster* have been conducted to find peptide precursor genes or genes of orphan G-protein-coupled receptor (GPCR), some of which were rediscovered as targets of intrinsic peptides [5].

Neuropeptide Y (NPY) in vertebrates, which has an amidated tyrosine residue, and neuropeptide F (NPF) in invertebrates, which has an amidated phenylalanine residue at the C terminus, are well conserved through evolution, and both are involved in feeding and/or foraging behaviors [6–9]. One type of *Drosophila* NPF consisting of 36 amino acid residues is referred to as long NPF [9, 10], whereas another type consisting of 6–11 amino acid residues is referred to as short NPF [9, 11]. Both the long and short NPFs are expressed in the brain and mid-gut of larvae and adults. Overexpression of long NPF prolongs the foraging period of larvae, resulting in a delay in the developmental sequence until pupation [12]. Short NPF is thought to be involved in food intake regulation during the larval stage, contributing to an increase in the body size [13].

Following the finding of receptors for these long and short NPFs, both of which show about 60 % sequence similarities to mammalian NPY receptors [11, 14], another GPCR, nepYR (CG5811), was identified in *D. melanogaster*. Although nepYR was activated by mammalian NPY [15], its intrinsic ligand was unknown until Ida et al. [16] discovered dRYamides-1 and -2. The precursor genes for these peptides (CG40733) are located in the centromeric heterochromatin of the right arm of chromosome 2, suggesting a low probability of construction of a transgenic fly bearing mutation in this chromosomal area. Thus, the same group explored the putative function of these peptides by injection into *P. regina*, which has long been used for physiological studies on the gustatory system or feeding behavior [17].

Flies detect various tastants with gustatory sensilla located on the labella at the distal end of proboscis, on the tarsi of legs, on the anterior wing margins, and on the ovipositor. The largest type of gustatory sensilla in *P. regina*, called an LL-type (200–300 μm in length), symmetrically lines the outer margin of labellar lobes and is one of the most precisely studied insect gustatory organs [17–24]. Each LL-type sensillum houses four gustatory receptor neurons (GRNs), and they respond to water, salts, bitter or noxious substances, and sugars or phagostimulative substances, respectively [17, 18, 20]. In several insect species, GRNs from the mouthparts and from the antennae directly project to the subesophageal ganglion (SEG) or gnathal ganglion (GNG) [25–28], which is the primary gustatory center in the fly brain [29, 30].

When the labellar gustatory sensilla are stimulated with sugar above a certain threshold concentration, flies extend their proboscides. PER is an early feeding behavior prior to food sucking and is an accepted indicator of feeding motivation in flies and some other insects [17, 20, 31]. In *P. regina*, it is known that the PER threshold is affected by starvation/satiation conditions [32], age and blood sugar level [33], daily dietary concentration of sugar [34], and experiences or learning about foods [35]. After completely extend the proboscis onto food, flies begin to feed. The termination of feeding is evoked under feedback regulation from stretch receptors in the foregut, and their activity may link to the sense of satiation [36–38].

The previous paper by Ida et al. [16] showed that 30 min after injection of 10 pmol dRYamide-1, the mean threshold of PER was significantly increased in a test population of *P. regina*. They conducted the PER test to investigate the effect of dRYamide-1 on feeding motivation of *P. regina* in one population but missed examination with dRYamide-2. Thus, in the present report we more precisely examine the involvement of these two peptides, dRYamides-1 and -2, in feeding regulation in three steps; food intake, proboscis extension reflex (PER), and sensory activation by phagostimulative taste.

## Materials and Methods

### Flies

*Phormia regina* (Diptera, Calliphoridae) blowflies were reared in our laboratory under 16-h light/8-h dark cycles at 21 ± 2 °C. Larvae were fed on chicken liver and yeast bait (Oriental Yeast, Tokyo, Japan). Newly emerged adults derived from the same egg masses were collected in separate plastic cages (22 × 15 × 13 cm^3^) and provided with water and 100 mM sucrose solution in separate cups. We used 5–7-day-old male flies in all experiments.

### Immunohistochemical procedure

For anti-dRYamide-1 antiserum production, we synthesized [Cys0]-dRYamide-1 peptide, which was then conjugated with keyhole limpet hemocyanin (KLH) (Medical and Biological Laboratories CO., LTD, Nagoya, Japan). Fly brains were dissected, immediately transferred into 4 % paraformaldehyde in phosphate buffered saline (PBS) containing 0.1 % Triton X-100, and incubated for two days at 4 °C. After rinsing three times for 10 min each in PBS containing 0.3 % Triton-X100, they were kept overnight at 4 °C, and with blocking solution of PBS plus 10 % goat serum for 2 h at room temperature. The brain samples were further incubated with primary antiserum at a dilution of 1:500 with blocking buffer for three days at 4 °C, followed by rinsing nine times for 20 min each in PBS plus 0.3 % Triton-X100. They were incubated with secondary antibody (Alexa Fluor 594 of goat anti-rabbit IgG) at a dilution of 1:200 with blocking buffer for two days at 4 °C, followed by rinsing nine times for 20 min each in PBS plus 0.3 % Triton-X100 and subsequently in Triton-X free PBS for 10 min. The brains were then dehydrated using an ethanol series (5 min in 50 %, 5 min in 70 %, 10 min in 90 %, 20 min in 100 % twice), and mounted with methyl salicylate. The samples were observed with a confocal laser-scanning microscope (FV1000 Olympus Co., Tokyo Japan).

### Peptide preparation and injection

Synthesis of dRYamides-1 and -2 was performed by Medical and Biological Laboratories (Nagano, Japan) according to the previously identified amino acid sequences PVFFVASRY-NH_2_ and NEHFFLGSRY-NH_2_, respectively [16]. These peptides were dissolved in a blowfly Ringer solution (128 mM NaCl, 5 mM KCl, 2 mM MgCl_2_, 1 mM Na_2_HPO_4_, 0.34 mM KH_2_PO_4_, 1.83 mM CaCl_2_, and 25 mM D-glucose) and diluted to 10 or 100 pmol/μL. Following this, 1 μL of Ringer solution or peptide-containing Ringer solution was injected into the shoulder (dorsal side of the thorax) of a test fly using a microsyringe (Hamilton Company, Nevada, USA) in accordance with Ida et al. [16]. As was learned from the previous work by Ida and his colleagues, artificial damage by injection frequently prevents flies from maintaining stable sensory responsiveness in gustatory receptor neurons and robust behavioral expression of feeding motivation or food intake for an hour or more, especially given the small body size of *D. melanogaster*. Thus, in the present study following the previous study by Ida et al. [16], we instead injected these peptides into *P. regina*, a larger fly species.

### Food intake measurement

Male *P. regina* were anesthetized on ice, and their wings were scarred with aluminum clothespins. In order to select individuals, which have average level of feeding motivation in a population reared for food intake measurement, the PER test described above was conducted. Individual flies, the PER threshold of which were equivalent to the mean threshold in the same batch population from the same egg mass, were next used for food intake measurement. The body weight of the selected flies was 26.4 ± 0.7 mg (average ± standard error). Among the selected individuals, we made random pairs having the smallest body weight difference (<1.5 mg).

**Fig. 1:**
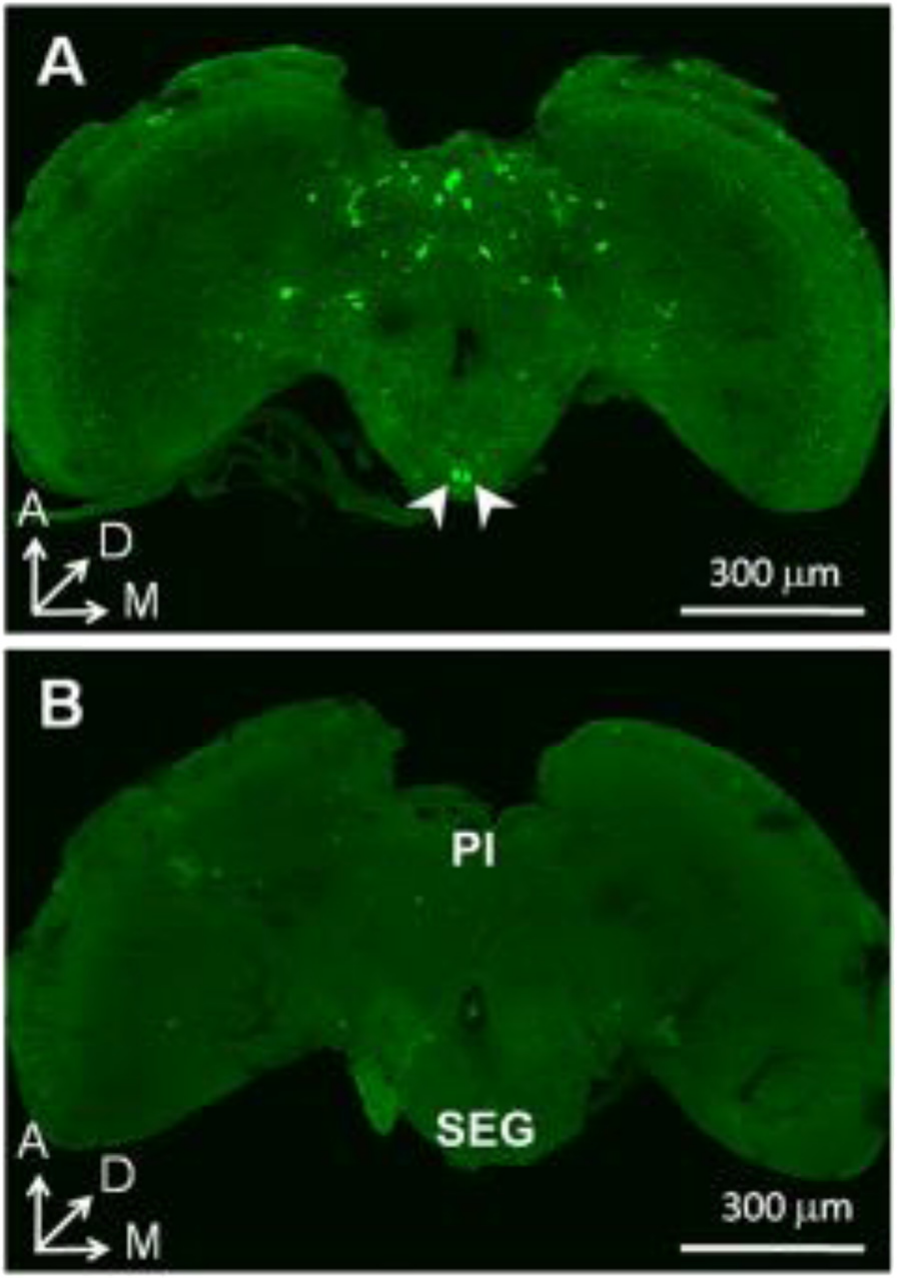
Immunohistochemical staining of the brain of P. regina with anti-dRYamide-1 antiserum. **a** Staining of male brain with anti-dRYamide-1 antiserum. **b** Same as (**a**) but treated with pre-immune serum instead of anti-dRYamide-1 antiserum. SEG, subesophageal ganglion/gnathal ganglion; PI, pars intercerebralis. Arrowheads indicate heavily stained cell bodies of two neurons (see Discussion)

We injected 1 μL of Ringer solution into one fly and the same volume of Ringer solution containing 0, 10, or 100 pmol dRYamide-1 or -2 into the other fly in each pair. Thirty minutes later, we started feeding the pair of flies sucrose solution at the same concentration, 250 mM, which is the mean PER threshold (see Fig. 4) or 1 M that is the concentration giving the maximum response of the labellar sugar receptor neuron [39]. We prepared two 25 μL drops of 250 mM or 1 M sucrose on a clean surface of Parafilm (Sigma-Aldrich Japan, Tokyo), and let the flies of a test pair contact with the drops in their labella, respectively. The flies then extended their proboscides and began feeding. Under constant ambient conditions (20–23 °C and 40–60 % relative humidity), we observed each pair of flies until the flies were satiated with the sugar solution and spontaneously stopped feeding. The intake volume of sucrose solution was measured by subtracting the leftover from the provided volume and compared between the Ringer solution-injected and the dRYamide-injected individuals.

### PER test

Flies extend their proboscides when stimulated by over-threshold phagostimulative tastants in their gustatory sensilla. The PER test to various concentrations of sucrose in a fly population has been conducted to determine the mean PER threshold concentration, which is a reciprocal indicator of feeding motivation [17, 20, 32. 34, 35]. The test fly population was starved for 24 h before the PER test. Prior to the PER test, 20 randomly chosen male flies were immobilized by securing their wings with aluminum clothespins and provided with distilled water until spontaneous satiation. For phagostimulative stimuli, 12 sucrose concentrations were prepared by twofold serial dilution with distilled water starting from 1 M. We carefully touched the labellar gustatory sensilla with each concentration of sucrose in a yellow micropipette tip, starting with the lowest concentration, and prevented the flies from ingesting the stimulus solution during the test. Subsequently, 1 μL of either Ringer solution or dRYamide-1-containing or dRYamide-2-containing Ringer solution was injected into a fly group of 20 individuals, and the PER test was repeatedly conducted until 60 min after the injection. Thus, we plotted the concentration–PER curves before and after the injection with the percentage of flies showing PER against the sucrose concentration.

### Electrophysiological procedures

Prior to the electrophysiological experiments, we selected individuals whose PER threshold was the same as the mean threshold in a test batch of flies, as described above. Under a microscope (Nikon ECLIPSE E200, Tokyo, Japan), the proboscis of each selected fly was fixed in an extended position with beeswax, and the body was immobilized by a stainless steel clip. Using a glass capillary containing a stimulus solution as the recording electrode, an LL-type labellar chemosensillum was stimulated for 1 s. A platinum wire inserted into the recording electrode was connected to a TastePROBE amplifier (Syntech, Hilversum, The Netherlands). Another glass capillary filled with Ringer solution functioned as an indifferent electrode was inserted into the compound eye. Impulses generated from the single sugar receptor neuron housed in the targeted sensillum were collected by a computer through an A/D converter, IDAC-4 (Syntech), and analyzed using Autospike software (Syntech).

Before and 15, 30, 45, and 60 min after the injection of 1 μL of either Ringer solution or dRYamide-1-containing or dRYamide-2-containing Ringer solution, impulses from the same sensilla were recorded using the tip-recording method [17, 40, 41]. Because the tip-recording method requires an electrolyte in stimulus solution filled in the recording electrode, we used a stimulus solution of 50 mM sucrose dissolved in 10 mM NaCl, which gives approximately 70 % of the maximum magnitude of response in the sugar receptor neuron [39]. This concentration of sucrose is convenient for detecting either positive or negative changes in the magnitude of response in the sugar receptor neuron. Because there are four functionally differentiated gustatory receptor neurons within a sensillum, when we stimulated the single LL-type sensillum using the tip-recording method, the sugar, salt, water, and bitter taste receptor neurons were all exposed to the stimulus. As long as 50 mM sucrose in 10 mM NaCl was used as a stimulus, however, the impulses of the sugar receptor neuron were mainly recorded. Sometimes, impulses of the water and/or the salt receptor neuron were also recorded, but these were only at low levels of frequency and could be easily distinguished from impulses of the sugar receptor neuron based on the difference in amplitude [40, 42]. The impulses of the sugar receptor neuron were selectively counted using an impulse sorting program (Syntech). According to nearly all electrophysiological reports for *P. regina* [39–41], the magnitude of response of the sugar receptor neuron can be defined as the number of impulses recorded for 0.2 s between 0.15 and 0.35 s after the beginning of stimulation, and it is kinetically changing according to the stoichiometric receptor-sugar binding reaction [40]. Signals were excluded during the initial 0.15 s, as a transient peak of impulse frequency in the sugar receptor neutron is not parallel with the gradual increase of the receptor membrane potential [41]. The impulses generated later than 0.35 s after the beginning of stimulation, the frequency of which is decreased by sensory adaptation [41], were also excluded from the definition of the magnitude of response.

## Results

### Localization of dRYamide-1 like peptide

As shown in Fig. 1a, the representative result in a male brain of *P. regina*, when stained with anti-dRYamide-1 antiserum, showed symmetrical staining of 26 cell bodies of neurons in the protocerebrum and pars intercerebralis and six cell bodies in SEG, two of which were heavily stained at the posterior-medial site (arrowheads). In addition, numerous button-like structures were stained in SEG. Figure 1b shows no specific staining in the brain treated with normal serum instead of anti-dRYamide-1 antiserum.

### No influence of dRYamides on food intake

**Fig. 2:**
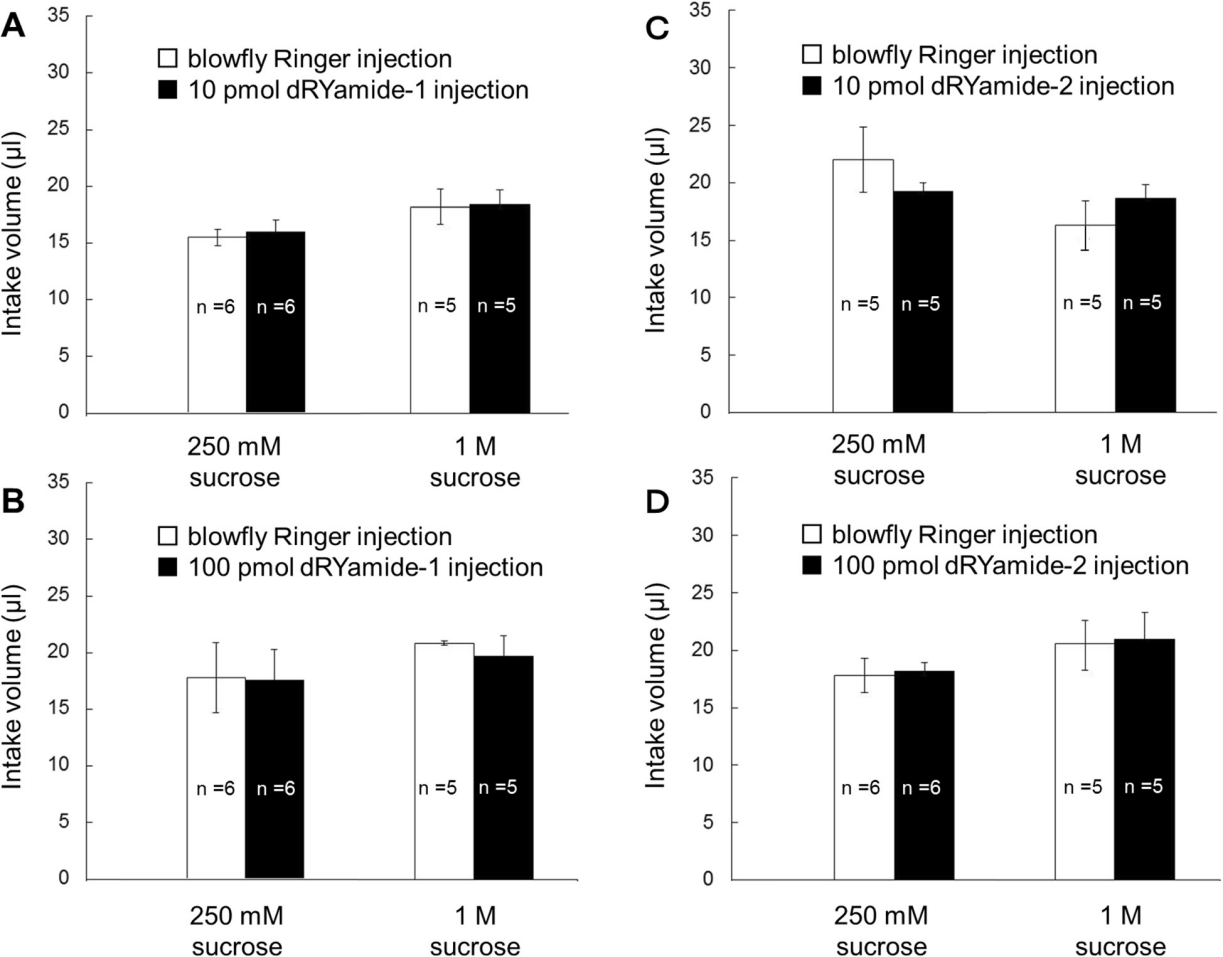
Effect of dRYamides-1 and -2 injection on sucrose intake. **a** Comparison of 250 mM or 1 M sucrose intake between blowfly Ringer solution-injected (open columns) and 10 pmol dRYamide-1-injected flies (closed columns). **b** Same as (**a**) but comparison between Ringer solution-injected (open columns) and 100 pmol dRYamide-1-injected flies (closed columns). **c** Comparison of 250 mM or 1 M sucrose intake between blowfly Ringer solution-injected (open columns) and 10 pmol dRYamide-2-injected flies (closed columns). **d** Same as (**c**) but comparison between Ringer solution-injected (open columns) and 100 pmol dRYamide-2-injected flies (closed columns. Each column indicates average intake volume of sucrose solution ± standard error (*n* = 5 or 6)

Figure 2 shows sucrose intake in the dRYamide-1 (Fig. 2a, b) or 2 injected flies (Fig. 2c, d), compared with that in control flies, which were injected with Ringer solution.

In the control experiments using Ringer solution-injected flies, when the intake volume was compared between 250 mM and 1 M sucrose, there was no significant difference in each case of Fig. 2a, b, c, and d (*P* >0.05, *n* = 5 or 6, Mann–Whitney *U* test). Moreover, even in the dRYamide-1-injected (Fig. 2a, b) or dRYamide-2-injected flies (Fig. 2c, d), intake volume of 250 mM or 1 M sucrose was not significantly changed from that in the Ringer solution-injected flies, regardless of the injection amount of dRYamides (*P* >0.5, *n* = 5 or 6, Mann–Whitney *U* test). Thus, dRYamide-1 or -2 was not involved in the mechanism for determination of intake volume of sucrose until spontaneous satiation.

### Suppressive effects of dRYamides on PER

**Fig. 3:**
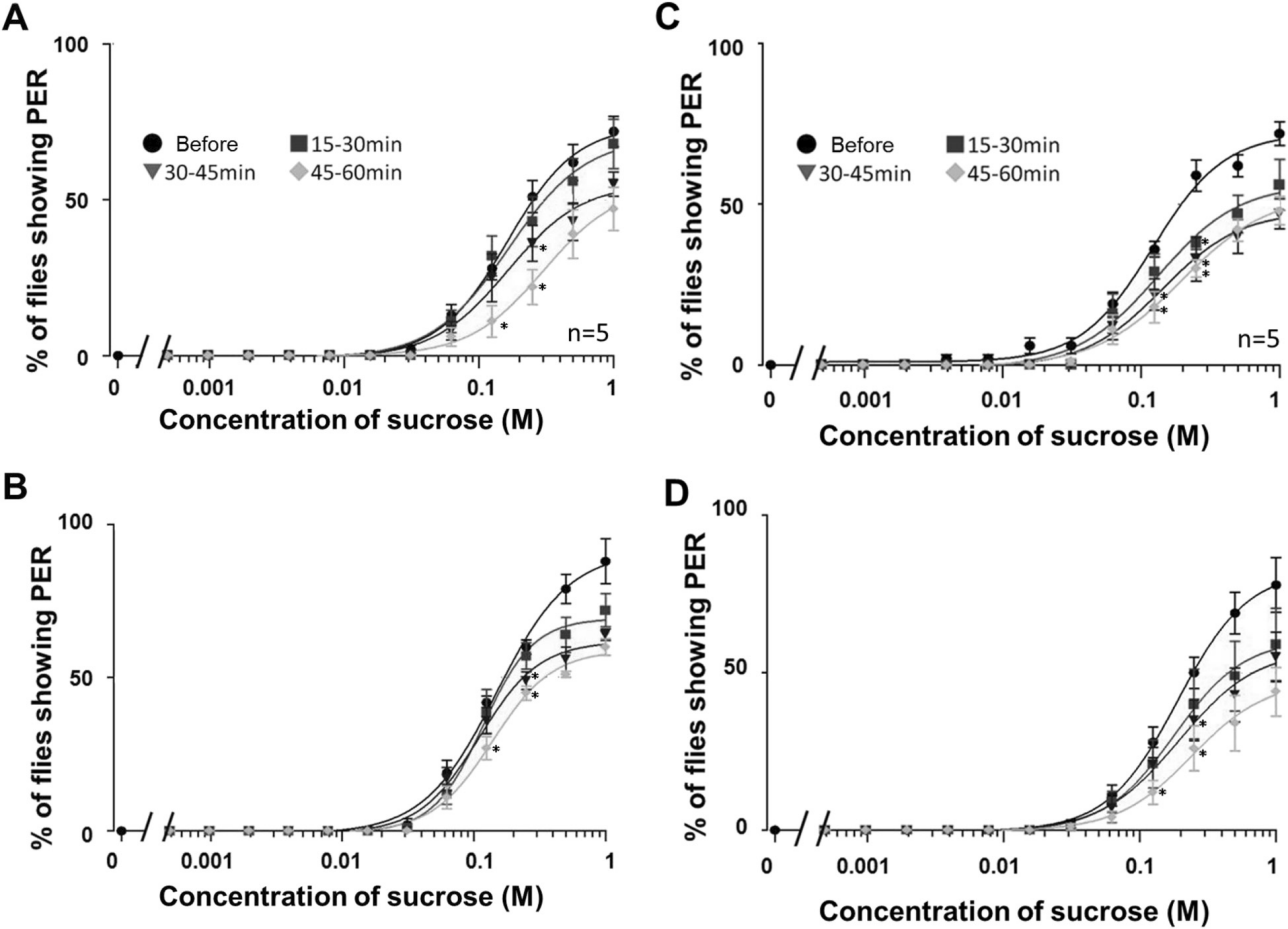
Suppressive effect of dRYamide-1 injection on PER. **a** The concentration–PER curves are drawn by plotting the percentage of flies showing PER (average ± standard error, *n* = 5) against stimulus sucrose concentration. **b** The same as (**a**) but the concentration–PER curves before and after 100 pmol dRYamide-1 injection. **c** The concentration–PER curves are drawn by plotting the percentage of flies showing PER (average ± standard error, *n* = 5) against stimulus sucrose concentration before and after 10 pmol dRYamide-2 injection. **d** The same as (**c**) but the concentration–PER curves before and after 100 pmol dRYamide-2 injection. Circles indicate data obtained before 10 pmol dRYamide-1 injection; squares, triangles, and diamonds indicate data obtained 15–30, 30–45, and 45–60 min after injection, respectively. Asterisks indicate statistically significant differences between the data collection intervals at given sucrose concentrations (*P* <0.05, *n* = 5, Mann–Whitney *U* test)

We performed PER tests before and after the injection of dRYamides-1 and -2 dissolved in the Ringer solution, respectively. Every concentration–PER curve was plotted for the percentage of flies showing PER against the sucrose concentration. Figure 3a, b show the series of the concentration–PER curves with the data obtained before and 15–30, 30–45, and 45–60 min after injection of 10 and 100 pmol dRYamide-1, respectively. Figure 3c, d are the same as Fig. 3a, b, respectively, but dRYamide-2 was injected instead of dRYamide-1. Both dRYamides-1 and -2, when injected into the thorax of flies, gradually suppressed PER to sucrose. After the injection of dRYamide-1 or -2, the percentage of flies showing PER was significantly decreased, as indicated by asterisks in Fig. 3 (*P* <0.05, *n* = 5, Mann–Whitney *U* test), but PER was not completely suppressed within 60 min. Regardless of 10 or 100 pmol of injected dRYamides, the percentage of flies showing PER to 1 M sucrose at 60 min after injection reached 60–70 % of that before injection.

**Fig. 4:**
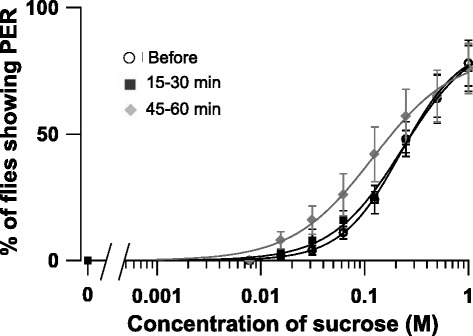
Effect of blowfly Ringer solution injection on PER. The concentration–PER curves are drawn by plotting the percentage of flies showing PER (average ± standard error, *n* = 5) against stimulus sucrose concentration. Open circles indicate data obtained before Ringer solution injection; squares and diamonds indicate data obtained 15–30 and 45–60 min after injection, respectively

With regard to the control experiment, in which Ringer solution was injected, there was no significant difference in the percentage of flies showing PER at any sucrose concentrations between before injection and 15–30 or 45–60 min after injection, (*P* >0.05, *n* = 5, Mann–Whitney *U* test) (Fig. 4). The PER ratio tends to increase at 45–60 min after Ringer injection in Fig. 4 for unknown reasons, but the increase does not reach statistical significance as defined in the present study.

### Suppressive effects of dRYamides on sugar taste response

**Fig. 5:**
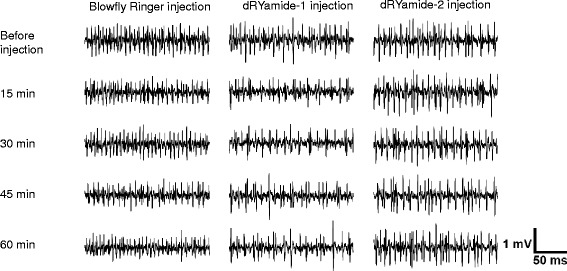
Impulse records of the sugar receptor neuron after injection of dRYamides Representative records of electrophysiological response of the sugar receptor neuron in the LL type of contact chemosensillum to 50 mM sucrose dissolved in 10 mM NaCl. (Left) Impulses in Ringer solution-injected fly; (Middle) impulses in the 10 pmol dRYamide-1-inected fly; (Right) impulses in the 10 pmol dRYamide-2-injected fly. Impulses were recorded before and 15, 30, 45, and 60 min after injection

**Fig. 6:**
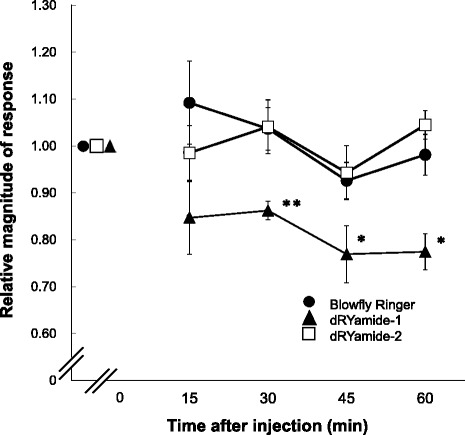
Effects of dRYamides-1 and -2 on the response of the sugar receptor neuron Changes in the magnitude of response of the sugar receptor neuron in the blowfly Ringer solution injected (closed circles), the 10 pmol dRYamide-1-injected (closed triangles), and the 10 pmol dRYamide-2-injected flies (open squares). Impulses were recorded before and 15, 30, 45, and 60 min after injection, and the magnitudes of response were normalized to those before injection. Single asterisks (*P* <0.05) and double asterisks indicate statistically significant differences (*P* <0.01, *n* = 5, post-hoc Tukey test)

Eleven pairs of LL-type sensilla of *P. regina* regularly align on the outer margin of both labellar lobes; therefore, we could easily identify every LL-type sensillum and repeatedly stimulate the same sensillum. Figure 5 shows representative impulse records before and 15, 30, 45, and 60 min after the injection of Ringer solution (left) or 10 pmol dRYamide-1-containing (middle) or 10 pmol dRYamide-2-containing Ringer solution (right). The electrophysiological activity with an impulse train generated from the sugar receptor neuron only decreased in dRYamide-1-injected flies. When we injected dRYamide-2 instead of dRYamide-1, no clear suppressive effect on the sugar taste response was observed. Figure 6 shows that the magnitude of response in the sugar receptor neuron gradually decreased to approximately 75 % of the response before the injection in dRYamide-1-injected flies only. The magnitude of response in the sugar receptor neuron was significantly different between the Ringer solution-injected and the dRYamide-1-injected flies at 30 (*P* <0.01, *n* = 5, post-hoc Tukey test), 45 (*P* <0.05, *n* = 5, post-hoc Tukey test), or 60 min after injection (*P* <0.05, *n* = 5, post-hoc Tukey test).

## Discussion

Studies in blowfly have long contributed to the understanding of feeding behavior and its neural mechanisms involving peripheral and central nervous systems [17, 20, 22, 32–38]. To date, many studies of the regulation of feeding and foraging behavior have been facilitated in *D. melanogaster* [7–15]. Nevertheless, the neural circuit from the phagostimulative gustatory input via the sugar receptor neuron to the behavioral output triggered by appropriate motor neurons has not been definitively identified. The neurons targeted by bioactive peptides related to feeding behavior and the steps of feeding behavior they directly or indirectly regulate remain unclear.

In our PER tests, we counted the number of flies showing full extension of proboscides, hence the flies showing PER were sometimes observed only about 75 % even on stimulation by 1 M sucrose (Figs. 3 and 4). If the flies showing halfway proboscis extension were also counted, the flies showing PER reached 100 % at 1 M, but we feel the approach we adopted here was superior for enabling precise evaluation. We found in the PER tests that the injection of dRYamide-1 or -2 partially but significantly suppressed PER (Fig. 3). This suggests that dRYamides negatively regulate feeding initiation in flies by suppressing feeding motivation. It is unclear why PER is not completely suppressed by these peptides. Although it may be technically difficult, if these peptides could be injected to the head instead of the thorax without inducing damage, they may act more rapidly and/or strongly on the PER-controlling neural system. These peptides are also expressed in the midgut and hindgut [16]; they may thus also be indirectly involved in feeding regulation via some route other than the neural circuit from the gustatory sensory input to the feeding motor neuron output.

After the proboscis is completely extended, the fly begins food intake. Our results of sucrose intake measurements showed that the flies finished sucrose sucking when the intake volume reached a certain level, regardless of the sucrose concentration. This implies that feeding termination does not depend on the nutritional value but on the intake volume of food, which can be monitored by the stretch receptor in the foregut [36, 37].

It has been reported that the injection of sulfakinin derived from *D. melanogaster*, DrmSKI to *P. regina* selectively suppressed the intake of carbohydrate [3], and it is considered that sulfakinin as well as short NPF [9–14] regulates feeding termination on the basis of the nutritional value of foods. Previously, leucokinin and its receptor were discovered in *D. melanogaster*, and found that the leucokinin pathway in *D. melanogaster* was suggested to be involved in a meal-size determination mechanism that is regulated by feeding termination via feedback signals from the stretch receptor [43].

In our experiment, notably, there was little difference in sucrose intake between Ringer solution-injected and either dRYamide-1- or -2-injected flies (Fig. 2), suggesting that feeding termination controlled by the feedback circuit from the foregut stretch receptor is not regulated by dRYamides. dRYamides negatively regulate feeding initiation or motivation, probably via another neural circuit from the gustatory neuron for triggering PER.

Because PER is triggered by over-threshold gustatory input from the sugar receptor neuron, gustatory sensitivity of the sugar receptor neuron is involved in PER sensitivity to sucrose. Our results showed that dRYamide-1 significantly suppressed responsiveness of the sugar receptor neuron but dRYamide-2 did not (Figs. 5 and 6), but the reason for this difference between the effects of dRYamide-1 and dRYamide-2 is unclear. Very recently, we purified a putative dRYamide-1-like peptide from the head of *P. regina*, which had a similar amino acid sequence (PSFFVGSRY-NH_2_) to dRYamide-1 (PVFFVASRY-NH_2_), but not to dRYamide-2 (NEHFFLGSRY-NH_2_); however, we could not find a dRYamide-2-like peptide in the crude head extract (unpublished data). Therefore, we suspect that *P. regina* may have an intrinsic receptor protein that binds more strongly to dRYamide-1 than to dRYamide-2. Such a putative receptor protein activated by dRYamide-1 may function in desensitization of the sugar receptor neuron. The receptor of dRYamides was discovered by Collin et al. [44]. They mentioned that activation of the *Drosophila* receptor by the two dRYamide peptides. The receptor, when experimentally expressed in culture cells, was activated within 5 s after addition of the peptides, after which the receptor quickly desensitizes to the zero level. On the other hand, our findings indicate that dRYamide-1 induced gradual desensitization of the sugar receptor cell and that the effect was incomplete, but lasted an hour (Fig. 6). Thus, we presume that dRYamide-1does not directly affect the sugar receptor neuron in *P. regina*; hence, its receptor, CG5811, would not be expressed in the sugar receptor neuron either in *D. melanogaster*. It has been reported that CG5811 is abundantly expressed in the hindgut, but not in the brain; however, we expect that the receptor of dRYamide-1 peptide is expressed in the brain, at least in the SEG region where the sugar receptor neurons terminate (see Fig. 1 and Additional file 1: Figure S1), As both dRYamide-1 and dRYamide-2, which was ineffective on desensitization of the sugar receptor neuron, similarly suppressed PER, it is unlikely that desensitization of the sugar receptor neuron alone causes PER suppression (Fig. 3). These peptides may also act on neurons other than the sugar receptor neuron. For example, they may affect putative interneurons including a command neuron [45, 46], whereby the PER threshold could be manipulated under various conditions such as the blood sugar concentration, trade off factors, and memory of dietary experiences.

Prior to our study, allatostatin-A of *D. melanogaster* was known to inhibit the PER in adult *D. melanogaster* [47]. The physiological function of allatostatin-A seems to be similar to that of dRYamides-1 and -2; however, the localization of anti-allatostatin-A antiserum-staining neurons in the brain of *D. melanogaster* [47] was not similar to that of anti-dRYamide-1 antiserum-staining neurons (Additional file 1: Figure S1). Our immunohistochemical data in Fig. 1 suggest that dRYamide-1 or presumably its ortholog of *P. regina* is likely to act on the primary gustatory center SEG, where gustatory information is transferred from the receptor neurons to some interneurons.

In the present study, we investigated the regulatory roles of dRYamides derived from *D. melanoaster* in feeding behavior of *P. regina*. Indeed, *D. melanogaster* and *P. regina* exhibit different habitats and food preferences, highlighting the need for additional study. In the near future, through further studies in *D. melanogaster* or through the use of a newly identified ortholog peptide in *P. regina*, the functional role and putative pathway of the dRYamide peptides will be confirmed.

## Conclusions

dRYamide-1 and dRYamide-2 were first reported as ligands of the neuropeptide Y-like receptor CG5811 in *Drosophila melanogaster*, and it has been suggested that dRYamide-1 suppressed the early feeding behavior, PER, in the blowfly *P. regina*. Starting from determining localization of dRYamide-1 in the brain of *P. regina*, we further investigated the regulatory roles of these peptides on the feeding behavior of *P. regina* in three steps: food intake, PER, and activity of the sugar receptor neuron. After injection of dRYamide-1 or -2, flies do not change their intake volume of sucrose solution, but a significant depression of PER to sucrose occurs. Injection of dRYamide-1 triggered a significant decrease in responsiveness of the sugar receptor neuron, while injection of dRYamide-2 does not. These results suggest that dRYamides decrease feeding motivation in flies and partially desensitize the sugar receptor neuron.

## Additional file

Additional file 1: Figure S1. Immunohistochemical staining of the brain of *D. melanogaster* with anti-dRYamide-1 antiserum. Arrow heads indicate heavily stained cell bodies of two neurons (see Discussion). (DOCX 476 kb)

## Abbreviations

PER: Proboscis extension reflex
GPCR: G-protein-coupled receptor
GRN: Gustatory receptor neuron
NPF: Neuropeptide F
NPY: Neuropeptide Y
PBS: Phosphate-buffered saline
SEG: Subesophageal ganglion
GNG: Gnathal ganglion
