# Supplementary material for: Suppressive effects of dRYamides on feeding behavior of the blowfly, Phormia regina
Source: Zoological Lett. 2015 Dec 8;1:35. doi: 10.1186/s40851-015-0034-z (PMC4672552; doi:10.1186/s40851-015-0034-z)
Supplement: Additional file 1: Figure S1. — Immunohistochemical staining of the brain of D. melanogaster with anti-dRYamide-1 antiserum. Arrow heads indicate heavily stained cell bodies of two neurons (see Discussion). (DOCX 476 kb) [file 40851_2015_34_MOESM1_ESM.docx]

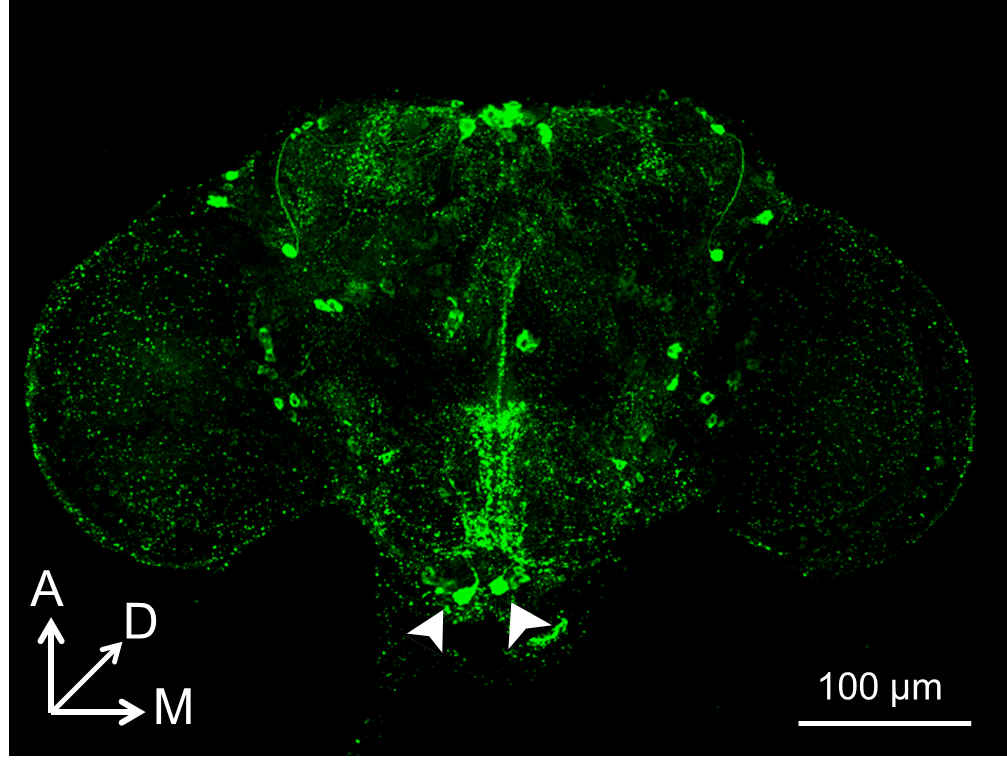


Figure S1 　Immunohistochemical staining of the brain of *D. melanogaster* with anti-dRYamide-1 antiserum. Arrow heads indicate heavily stained cell bodies of two neurons (see Discussion).
